# Trichosporon Species and Fusarium Species as a Cause of Empyema Thoracis in a Diabetic Patient

**DOI:** 10.7759/cureus.8973

**Published:** 2020-07-02

**Authors:** Nousheen Iqbal, Muhammad Ammar, Muhammad Irfan, Kauser Jabeen

**Affiliations:** 1 Section of Pulmonary and Critical Care Medicine, Aga Khan University, Karachi, PAK; 2 Medicine / Pulmnonology, Jinnah Medical and Dental College, Karachi, PAK; 3 Internal Medicine, Dow International Medical College, Karachi, PAK; 4 Internal Medicine, Aga Khan University, Karachi, PAK; 5 Pathology, Aga Khan University, Karachi, PAK

**Keywords:** diabetes, fungal empyema, trichosporon species, fusarium species

## Abstract

Of late, fungal infections are increasingly being recognized in diabetic patients. Here we present a case of polymicrobial fungal empyema due to Trichosporon species and Fusarium species developed after community-acquired pneumonia in a diabetic patient. Trichosporon species are basidiomycetous yeast and Fusarium species are soil saprophytes with a worldwide distribution. Fungal empyema cases are rare and are mostly caused by Aspergillus and Candida species. Polymicrobial fungal empyema with Trichosporon species and Fusarium species has not been reported previously. Our patient was successfully treated with antifungal therapy. This case highlights that fungal empyema should be considered in diabetic patients especially if they are not responding to antibiotics.

## Introduction

Empyema is mainly caused by bacterial agents; *Streptococcus* *milleri*, *S. viridans*, and *S. pneumoniae* being the most common agents in community-acquired empyema [1]. Empyema is rarely caused by fungi and *Aspergillus* species and *Candida* species are frequently reported agents [2]. Risk factors for fungal empyema include diabetes, immunosuppression, invasive surgical procedures with nonsterile equipments, and esophago- or gastro-pleural fistula [3-6]. Fungal infections have been increasingly recognized in diabetic patients because of their altered immune status [7].

*Trichosporon* species are basidiomycetous yeast-like anamorphic pathogens which are generally distributed in nature and found mainly in tropical and temperate areas. These organisms can be found in soil, decomposing wood, air, rivers, lakes, sea water, cheese, cattle, scarab beetles, pigeons, and bird droppings [8-10]. It can cause invasive infections in immunocompromised patients whereas in immunocompetent hosts it results in allergic pneumonia and superficial infections [11].

*Fusarium* species are soil saprophytes with a worldwide distribution and result in a broad spectrum of infections, ranging from superficial to locally invasive or disseminated infections. Severely immunocompromised patients suffer from disseminated infection while immunocompetent individuals have allergic diseases and other disease manifestations [12-13].

Here we are reporting a case of a diabetic patient who developed empyema with *Trichosporon* species and *Fusarium* species after community-acquired pneumonia. The patient was treated successfully with antifungal agents. This case highlights the significance of super-added fungal infection as a cause of empyema in a diabetic patient with community-acquired pneumonia.

## Case presentation

A 63-year old female was referred to our hospital with progressively increasing shortness of breath for 15 days. She was being treated as a community-acquired pneumonia patient at some other hospital. Her course of illness at that hospital was complicated with the development of right-sided hydropneumothorax, for which she had underwent right-sided tube thoracostomy. Her condition did not improve, and she was referred to our hospital. On presentation her blood pressure was 110/60 mmHg, pulse was 110/min, and oxygen saturation was 95% on 10 L of oxygen. She had severe subcutaneous emphysema on face and chest. There was a chest tube placed on the right side. On chest auscultation bilateral crackles were audible. Rest of the examination was unremarkable. She was a known case of hypertension and poorly controlled diabetes mellitus, her glycated hemoglobin (HbA1c) on admission was 8.7%. She was on metformin and Humulin N. During hospitalization her blood sugar remained elevated for which Insulin Glargin 24 units was added with increased dose of Humulin N. There was no prior history of diabetic neuropathy, retinopathy, or nephropathy.

**Figure 1:**
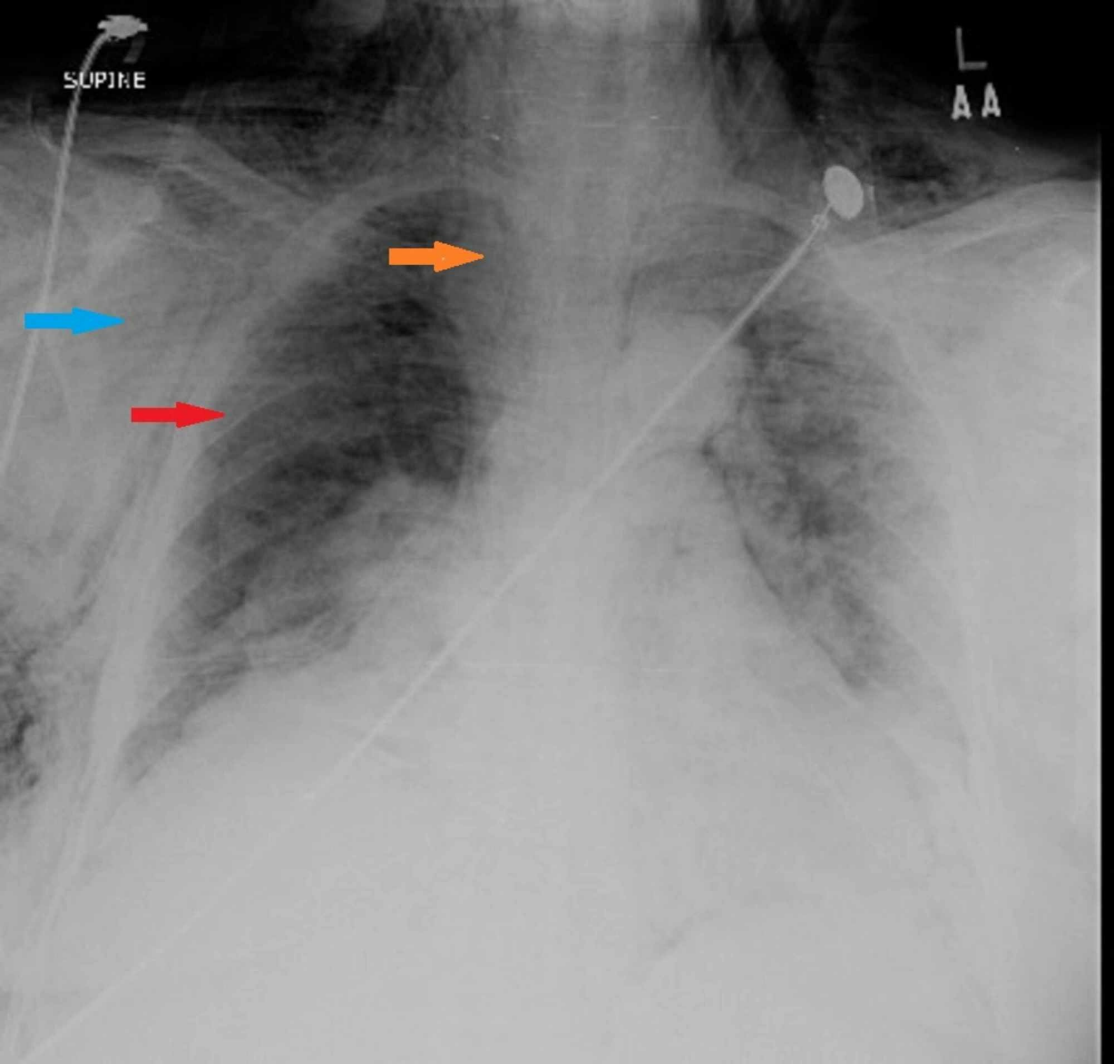
Chest X-ray showing bilateral subcutaneous emphysema (blue arrow), pneumomediastinum (orange arrow), and right-sided pneumothorax (red arrow).

**Figure 2:**
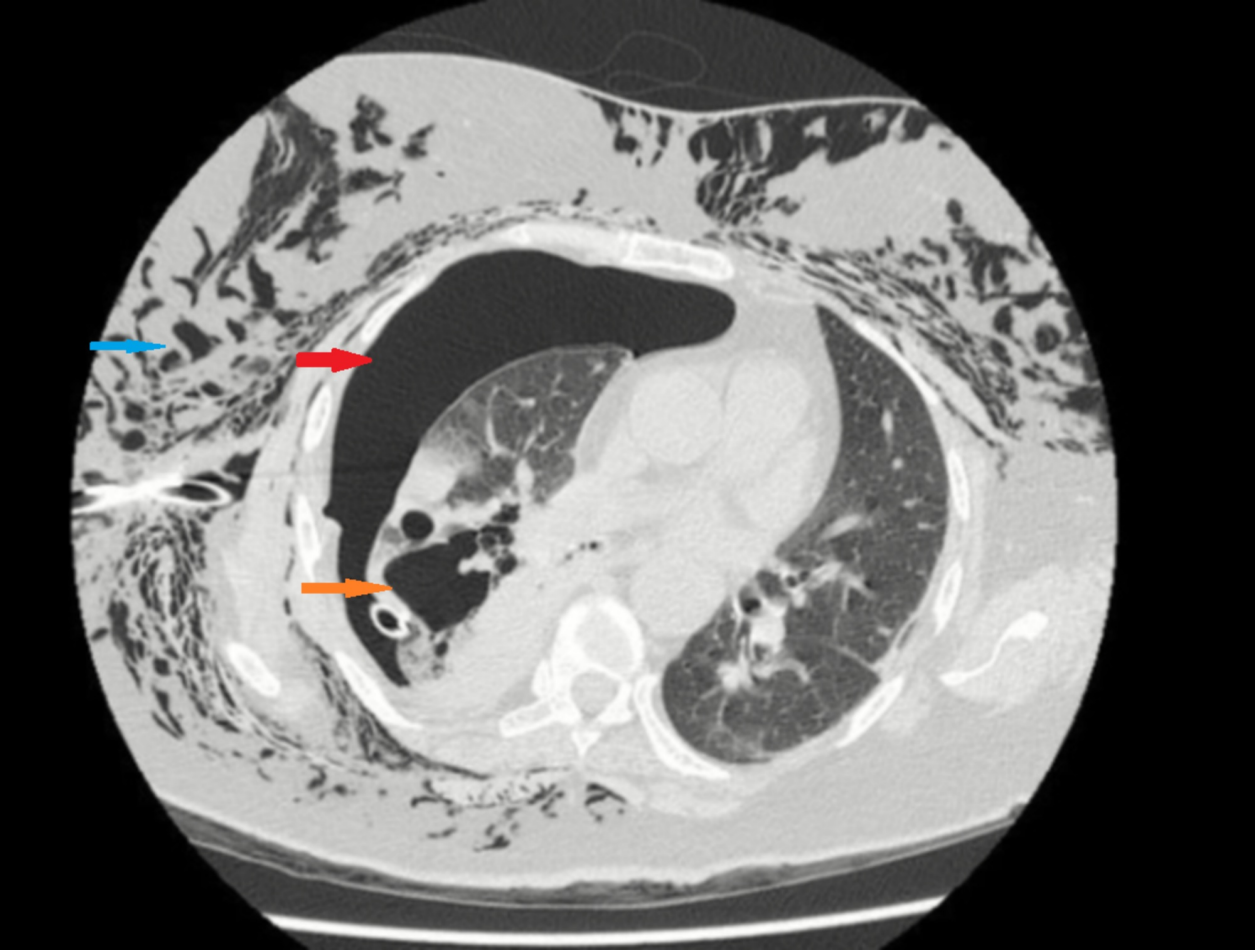
High resolution CT chest showing right cavitation (orange arrow) with pneumothorax (red arrow) and subcutaneous emphysema (blue arrow).

On presentation her complete blood count showed hemoglobin of 12.7 g/dL and white cell count of 14.8 x 103/L with 84.4% neutrophils. Blood urea nitrogen was 15 mg/dL, creatinine was 1.0 mg/dL, and random blood sugar was 294 mg/dL. Chest radiograph revealed significant subcutaneous emphysema with right-sided pneumothorax, pneumomediastinum along with pneumopericardium (Figure 1). High resolution computed tomography (HRCT) scan confirmed pneumothorax with pneumomediastinum, pneumopericardium, right-sided necrotizing consolidation, and mal-positioning of chest tube (Figure 2).

The patient was started on piperacillin/tazobactam, vancomycin, and a new chest tube was inserted on the right side. Due to no improvement in her condition, surgical intervention was planned. The patient refused surgical intervention and was discharged home on IV antibiotics with chest tube on suction. She again presented 10 days later with fever and purulent discharge from the chest tube. Pus was sent to laboratory for Gram stain and culture. Antibiotics were changed to colistin and meropenem. Pleural fluid analysis showed total leukocyte count of 99020/cu mm with 90% polymorphs and glucose of 7 mg/dL. Gram stain of pleural pus revealed numerous pus cells and fungal hyphae on smear and culture grew *Trichosporon* spp. and *Fusarium* spp. *Fusarium* spp. was identified based on phenotypic gross and microscopic morphology, while *Trichosporon* spp. was identified to genus level based on colony morphology, urease production, and microscopic morphology on corn meal agar. Susceptibility testing for both organisms was not performed. No bacterial growth was obtained on sputum, blood, and pleural culture. As tuberculosis is endemic in Pakistan, she was also evaluated for TB and mycobacterial culture was negative.

**Figure 3:**
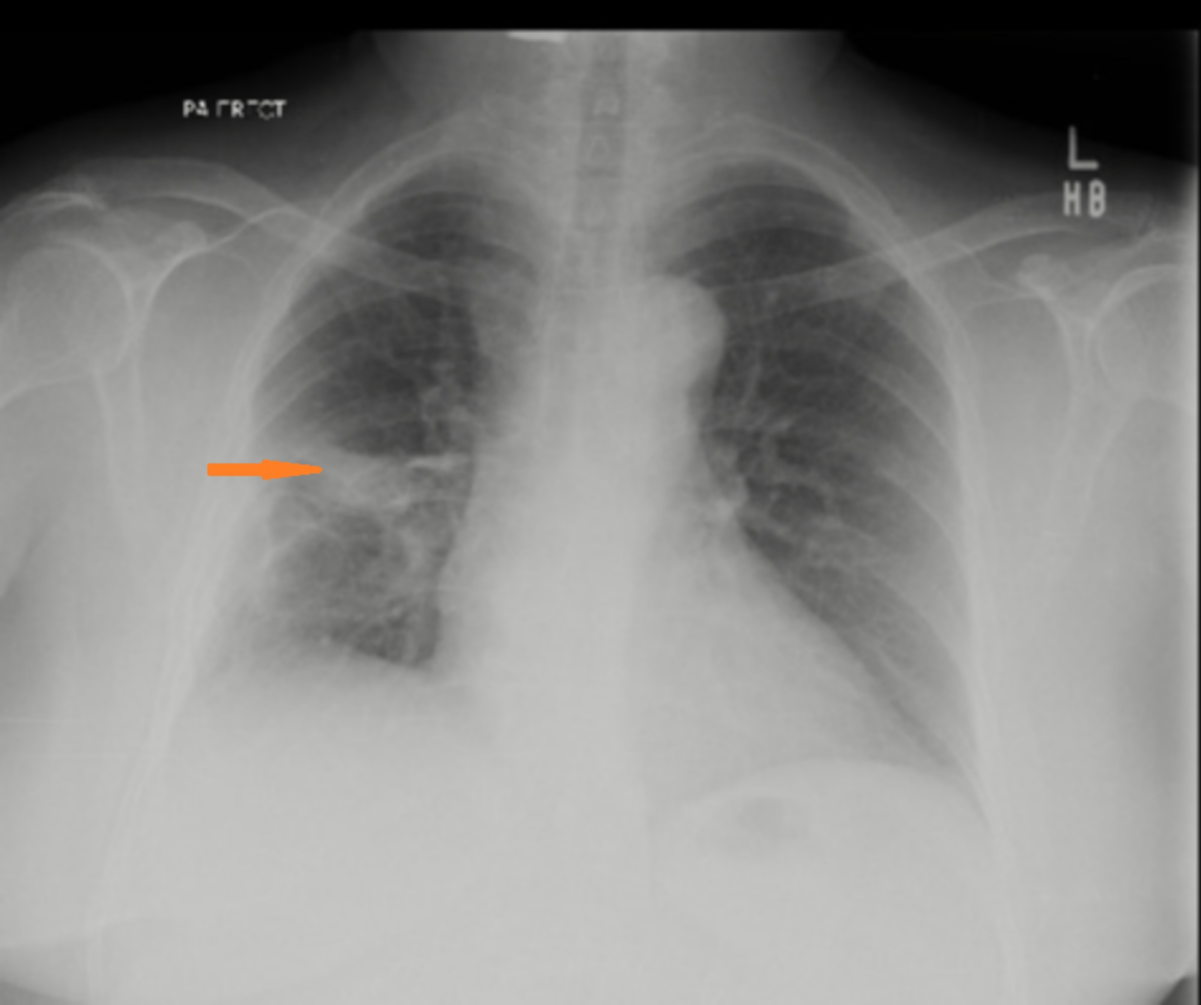
Chest X-ray after treatment showing fibrotic changes (arrow) at right middle zone.

Amphotericin B deoxycholate was started and antibiotics were stopped after the third day of admission. Empyema was drained through chest tube and the chest tube was kept in place for eight weeks. She became afebrile after initiation of antifungal but developed acute kidney injury, electrolyte imbalance, and vomiting. Amphotericin B was replaced with voriconazole 200 mg twice daily which was continued for eight weeks. She tolerated the treatment very well and her condition improved gradually, and the chest tube was successfully removed after eight weeks. Follow up chest radiograph showed significant improvement (Figure 3).

## Discussion

Fungal empyema itself is a rare entity with *Aspergillus* spp. and *Candida* spp. as the most commonly reported organisms [2-6]. Although isolated cases of empyema with *Fusarium* spp. and *Trichosporon* spp. have been reported co-infection with both organisms has not been reported previously [14]. Fungal empyema has been reported mainly in patients with immunosuppression, brochopulmonary infection, and postabdominal or chest surgery. Mortality in these cases remains high if not diagnosed and left untreated. Our patient was treated successfully with antifungal without any surgical intervention.

Treatment of empyema could be surgical with video-assisted thoracoscopic surgery (VATS) or open thoracotomy and nonsurgical such as thoracentesis and chest tube drainage with or without fibrinolytic agents [15]. Despite receiving surgery and pleural irrigation with antifungal agents the crude mortality rate has been reported to be as higher as 73% in fungal empyema [2]. Treatment of fungal empyema is not standardized and several drugs can be used [16]. Our patient refused surgical intervention so she was managed with chest tube drainage and systemic antifungal therapy.

*Trichosporon* can colonize different parts of human body. This pathogen is also able to cause deep-seated, mucosa-associated, or superficial infections. Invasive trichosporonosis has been observed commonly in immunosuppressed patients or with hematological malignancies and in critically ill patients who have been exposed to multiple invasive medical procedures [8-10]. Similarly *Fusarium* can also be a colonizer but can cause invasive infection in immunocompromised host [12]. Treatment of both of these fungi is species specific with some of the species having higher minimum inhibitory concentrations with amphotericin or azoles. We identified these organisms up to genus level only and susceptibility testing was also not performed.

Our patient had no other apparent risk factor of fungal infection except diabetes. However, the patient had had ICU admission, underwent invasive procedure like chest tube insertion, and received multiple broad spectrum antibiotics; all of which could increase the risk of invasive fungal infection. The other possibility is colonization but our patient did not grow any bacteria throughout her illness and did not show any improvement despite being on broad spectrum antibiotics. The patient showed significant improvement after adding antifungal therapy. This suggests that these organisms were pathogens and not a colonizer in our patient.

## Conclusions

Our case highlights the importance of super-added fungal infection especially if patients do not respond to the conventional antibiotic therapy. Failure of treatment should raise the suspicion of other organisms as diabetic patients are at higher risk of not only bacterial but also of fungal infections. Early diagnosis and management lead to good clinical outcome.
